# Effectiveness of acupuncture as auxiliary combined with Western medicine for epilepsy: a systematic review and meta-analysis

**DOI:** 10.3389/fnins.2023.1203231

**Published:** 2023-07-20

**Authors:** Hua Xue, Li Zeng, Hongxian He, Dongxun Xu, Kaixin Ren

**Affiliations:** ^1^Department of Neurology, Sichuan Taikang Hospital, Chengdu, Sichuan, China; ^2^Department of Respiratory, Affiliated Hospital of Youjiang Medical University for Nationalities, Baise, Guangxi, China; ^3^Department of Rehabilitation, Affiliated Hospital of Yunnan University, Kunming, Yunnan, China

**Keywords:** acupuncture, epilepsy, meta-analysis, randomized controlled trials, systematic review

## Abstract

**Background:**

Although more and more clinical studies have shown that acupuncture as an auxiliary combined with Western medicine is effective in the treatment of patients with epilepsy, no systematic reviews of acupuncture as a treatment for epilepsy have been published. Hence, we conducted this meta-analysis to evaluate the effect of acupuncture treatment on patients with epilepsy.

**Methods:**

This study retrieved randomized controlled trials (RCTs) of acupuncture treatment for epilepsy from various electronic databases including PubMed, Embase, Cochrane Library, China National Knowledge Infrastructure, Chinese BioMedical Literature Database, and Wangfang database. These studies evaluated the effectiveness of acupuncture as an auxiliary treatment combined with Western medicine for patients with epilepsy. The methodological quality of the studies was assessed using the Cochrane Handbook for Systematic Reviews of Interventions.

**Results:**

A total of 17 RCTs involving a total of 1,389 participants were included. The results showed that acupuncture combined with Western medicine improved the effective rates of treatment (OR: 4.28; 95% CI: 3.04–6.02; *p* < 0.001), and reduced the seizure frequency of patients (SMD: −3.29; 95% CI: −3.51 to −3.07; *p* < 0.001) and the EEG discharge frequency (SMD: −5.58; 95% CI: −7.02 to −4.14; *p* < 0.001). Regarding the quality of life and adverse events, the acupuncture group was superior to the control group in improving the overall quality of life of patients with epilepsy (SMD: 14.41; 95% CI: 12.51–16.32; *p* < 0.001) and decreased adverse events (OR: 0.38; 95% CI: 0.23–0.63, *p* < 0.001).

**Conclusion:**

The results of the analysis suggested that acupuncture combined with Western medicine is probably helpful in patients with epilepsy, but strong supportive data are not yet available. Given that this study is based on a low to moderate evidence-based analysis, the conclusions should be viewed with caution.

**Systematic review registration:**

PROSPERO, identifier no. CRD42023409923.

## 1. Introduction

Epilepsy is a neurological disease caused by abnormal discharge of nerve cells in the brain caused by various reasons (Chen et al., 2018). The main clinical manifestations are transient disturbance of consciousness, limb twitching, sensory or behavioral disturbance, and autonomic dysfunction (Yang et al., 2018). The number of epilepsy patients worldwide accounts for 0.5–0.7% of the total population (Orsini et al., 2020). There are nearly 10 million epilepsy patients in China, and the number is gradually increasing. About 10% of people will experience a seizure in their lifetime (Knowles et al., 2022). Currently, epilepsy can be treated with anti-seizures medications (ASMs) or surgery (Rugg-Gunn et al., 2020). Commonly used ASMs include carbamazepine, sodium valproate, benzodiazepines, lamotrigine, and oxcarbazepine (Vidaurre et al., 2017). ASMs control epileptic seizures by regulating voltage-gated sodium channels, voltage-gated calcium channels, and blocking ionotropic glutamate receptors (Manford, 2017). Although ASMs treatment can quickly relieve patients’ discomfort symptoms and reduce the damage to the brain caused by seizures in patients with epilepsy, there are many adverse reactions of ASMs, such as drowsiness, dizziness, nausea, vomiting, and even severe liver failure and renal function damage (Kanner et al., 2018). On the other hand, ASMs should take a long time, resulting in poor medication compliance, which is not conducive to the cure of epilepsy, thus seriously affecting the quality of life of patients with epilepsy.

In China, more and more clinical studies suggest that acupuncture combined with drug treatment for epilepsy has achieved efficacy, with the advantages of low side effects and simple operation, and these advantages make up for the shortcomings of Western medicine for epilepsy to some extent (Sun, 2018; Oliveira et al., 2021). Therefore, in this study, the effectiveness and safety of acupuncture combined with Western medicine in the treatment of epilepsy were investigated and analyzed from the aspects of effects and adverse reactions after acupuncture combined with Western medicine, and the effectiveness and safety of acupuncture combined with Western medicine in the treatment of epilepsy were systematically evaluated by Meta-analysis, in order to provide evidence-based medical evidence for clinical treatment.

## 2. Methods

### 2.1. Search strategy and selection criteria

This systematic review and meta-analysis are fully compliant with the Preferred Reporting Items for Systematic Reviews and Meta-analysis (Page et al., 2021). A detailed protocol for this study was registered at PROSPERO (no. CRD42023409923).

The randomized controlled trials (RCTs) of acupuncture as an adjunct to Western medicine in the treatment of patients with epilepsy was considered eligible in our Meta-analysis, and the language of publication was limited to English and Chinese. The following electronic databases were systematically and comprehensively searched: PubMed, Embase, Cochrane Library, China National Knowledge Infrastructure (CNKI), China Biomedical Literature Database (CBM), and Wanfang Database. The retrieval time is from the establishment of the database to April 2023. Search following keywords included “acupuncture,” “acupuncture treatment,” “needle,” “epilepsy,” “randomized controlled trial,” and “clinical trials.” The complete search strategy is shown in Supplementary material. In addition, we conducted a manual search of the reference list of the retrieved studies to identify other potentially eligible studies. Two reviewers independently selected the studies, and if there were differences, they were resolved through a third-reviewer discussion.

The study was included in our systematic review if the following criteria were met: (1) Patients diagnosed with primary epilepsy had to meet available diagnostic criteria such as the “2000 Chinese Medical Association recommendations for diagnosis and treatment of epilepsy,” “International League Against Epilepsy 2014 (ILAE),” “the 10th revision of International Classification of Diseases (ICD-10)” and other standards or consensuses. Patients with post-stroke epilepsy, post-traumatic epilepsy, post-operative epilepsy, etc. will be excluded; (2) Intervention: patients received acupuncture treatment or acupuncture combined with drug treatment; (3) Control: patients received conventional Western medicine treatment; (4) Outcome: effective rate, adverse events, seizure frequency and Quality of Life Scale for Epileptic Patients-31(QOLIE-31); (5) Study design: only RCTs were considered. Quasi-RCTs, animal studies, case reports, full text not available or no data available, and review articles were excluded.

### 2.2. Data collection and quality assessment

Two reviewers independently extracted the following information from included studies: first authors’ name, publication year, study design, sample size, mean age, disease duration, male proportion, intervention and control, follow-up duration, and reported outcomes. Any inconsistent results concerning data collection between reviewers were resolved by group discussion until a consensus was reached. The quality of the included literature was evaluated by two reviewers independently using Version 2 of the Cochrane risk-of-bias assessment tool (ROB 2). The assessment tool focused on the randomisation process, deviations from the intended interventions, missing outcome data, measurement of the outcome, selection of the reported result, and other bias. Each entry was judged to be: low risk, high risk, or some concerns. Any discrepancies regarding the assessment were resolved through discussion with a third investigators.

### 2.3. Outcome measures

The outcome measures were (1) effective rate, (2) adverse events, (3) seizure frequency, (4) Electroencephalogram (EEG) related indicators, including EEG discharge frequency and the power of brain wave in EEG (α wave, β wave, δ wave and θ wave) and (5) Quality of Life Scale for Epileptic Patients-31(QOLIE-31). After treatment, the clinical symptoms of the patients showed improvement and the frequency of seizures decreased by 50% or more, indicating effectiveness. The effective rate was regarded as a dichotomous measure (effective or ineffective). The QOLIE-31 is a scale that assesses the ability to live with epilepsy. It has 31 items, divided into seven dimensions and one overall item, with higher scores representing a higher quality of life, out of 100.

### 2.4. Statistical analysis

We used Review Manager (RevMan) software version 5.3.0 (Cochrane Central Executive Team, United Kingdom) as recommended by Cochrane Collaboration to process statistical analysis. Heterogeneity was measured using both Cochran’s *Q*-test (*p* ≤ 0.10 was used to define a significant degree of heterogeneity) and the *I*^2^ statistic (*I*^2^ > 50% showed the existence of heterogeneity). If the heterogeneity was significant (*p* < 0.1, *I*^2^ > 50%), a random effect (RE) model was chosen to pool the data, and if there was acceptable heterogeneity (*p* ≥ 0.1, *I*^2^ ≤ 50%), a fixed effect (FE) model was used. The mean difference (MD) or standard mean difference (SMD) was used to represent continuous data, and the Odds Ratio (OR) were used to represent dichotomous data. Results were reported with 95% confidence intervals (CI), and *p* < 0.05 was considered a significant statistical effect.

## 3. Results

### 3.1. Searching published literature and study characteristics

**Figure 1:**
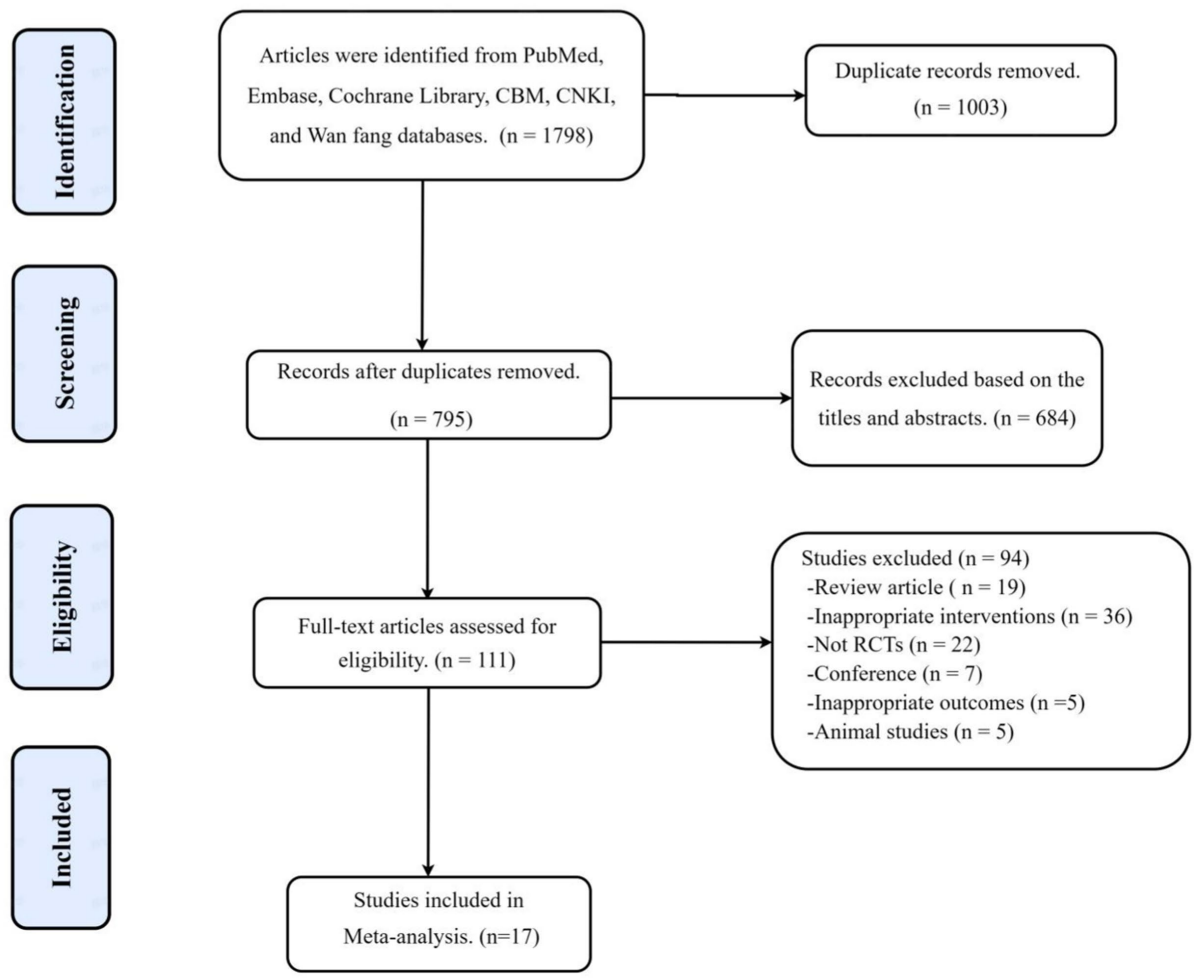
Flowchart of trial selection process for this systematic review.

A total of 1,798 articles were searched according to the search strategy, 1,003 duplicate references were excluded, 684 irrelevant articles were excluded by reading the title and abstract, and 111 articles were retrieved for further evaluation. A total of 94 articles were further excluded because of not randomized controlled trials (*n* = 22), inappropriate interventions (*n* = 36), not human (*n* = 5), review articles (*n* = 19), and conference (*n* = 7). Finally, a total of 17 studies were selected for the final meta-analysis (Figure 1).

The basic information of the included studies is shown in Table 1 (Niu, 2014; Yu, 2015; Zhang et al., 2017; Liu, 2018; Wang M., 2018; Wang Z. J., 2018; Zhu, 2018; Huang and Liu, 2020; Li, 2020; Shang, 2020; Zhang, 2020; Zhou, 2021; Liu, 2022; Ma et al., 2023; Wang et al., 2023; Xin et al., 2023; Ye, 2023). 17 RCTs with a total of 1,389 patients were included, including 693 in the experimental group and 696 in the control group. 17 RCTs published between 2014 and 2023, with a minimum sample size of 40 participants and a maximum of 160 participants, were included in this review. Basic information about studies included first author, publication years, study design, sample size, mean age, disease duration, male ratio, interventions, controls, treatment duration, follow-up time, and outcome indicators. All studies assessed the effective rate of treatment, seven articles assessed adverse effects, eight articles assessed seizure frequency, three RCTs recorded EEG related indicators, and only three articles assessed QOLIE-31.

**Table 1:** Characteristics of included studies and patients.

Study	Study design	Sample size (T:C)	Age (year), mean ± SD	Disease duration (year), Mean ± SD	Sex (M:F)	Intervention	Comparison	Treatment duration (months)	Follow-up (months)	Outcomes
Huang et al., 2020	RCT	55:55	T: 43.68 ± 6.83 C: 43.76 ± 6.74	T: 3.91 ± 1.68 C: 3.15 ± 1.51	(T) 35:20 (C) 36:19	Sodium valproate, acupuncture	Sodium valproate	3	NM	Effective rate
Li et al., 2020	RCT	41:41	T: 46.4 ± 4.5 C: 46.2 ± 4.3	NM	(T) 23:18 (C) 24:17	Sodium valproate, acupuncture	Sodium valproate	2	6	Effective rate Adverse events
Liu et al., 2018	RCT	60:60	T: 30.9 ± 9.7 C: 31.2 ± 9.6	NM	(T) 22:38 (C) 24:36	Topiramate, acupuncture	Topiramate	NM	NM	Effective rate Adverse events
Liu et al., 2022	RCT	30:30	T: 45.6 ± 6.4 C: 45.8 ± 6.3	T: 4.8 ± 0.9 C: 4.9 ± 0.8	(T) 13:17 (C) 14:16	Sodium valproate, acupuncture, ditan-tongqiao-xingnao decoction	Sodium valproate	3	NM	Effective rate Seizure frequency MoCA MMSE QOLIE-31
Ma et al., 2023	RCT	45:45	T: 40.15 ± 5.26 C: 41.05 ± 5.33	T: 1–5 C: 1–6	(T) 28:17 (C) 30:15	Levetiracetam, acupuncture	Levetiracetam	3	NM	Effective rate
Niu et al., 2014	RCT	30:30	T: 35.3 ± 8.5 C: 33.3 ± 7.9	T: 4.6 ± 2.4 C: 4.3 ± 2.6	(T) 13:17 (C) 15:15	Sodium valproate, acupuncture	Sodium valproate	3	NM	Effective rate
Shang et al., 2020	RCT	41:41	T: 39.42 ± 5.18 C: 39.07 ± 5.35	T: 2.30 ± 0.59 C: 2.19 ± 0.63	(T) 24:17 (C) 22:19	Topiramate, acupuncture, qufeng-dingxian decoction	Topiramate	6	NM	Effective rate Seizure frequency EEG discharge frequency
Wang et al., 2018	RCT	30:30	T: 32.5 ± 6.5 C: 31.9 ± 7.1	T: 6.3 ± 2.4 C: 7.1 ± 1.9	(T) 14:16 (C) 15:15	Sodium valproate, acupuncture	Sodium valproate	1	NM	Effective rate Seizure frequency QOLIE-31
Wang et al., 2018	RCT	40:40	T: 18.3 ± 4.0 C: 18.1 ± 3.8	NM	(T) 23:17 (C) 22:18	Sodium valproate, acupuncture	Sodium valproate	1	NM	Effective rate Seizure frequency HAMD Adverse events
Wang et al., 2023	RCT	56:57	T: 7 ± 1 C: 7 ± 1	T: 2.01 ± 1.03 C: 1.97 ± 0.94	(T) 24:32 (C) 34:23	Sodium valproate, acupuncture	Sodium valproate	3	4	Effective rate Seizure frequency
Xin et al., 2023	RCT	80:80	T: 30.05 ± 8.11 C: 29.79 ± 8.24	NM	(T) 43:37 (C) 36:44	Sodium valproate, acupuncture	Sodium valproate	1	NM	Effective rate Seizure frequency Adverse events
Zhang et al., 2020	RCT	30:30	T: 54.1 ± 5.13 C: 53.5 ± 5.47	NM	(T) 12:18 (C) 13:17	Sodium valproate, oxcarbazepine, acupuncture	Sodium valproate, oxcarbazepine	1	1	Effective rate Seizure frequency
Zhang et al., 2017	RCT	20:20	T: 45 ± 5 C: 43 ± 5	NM	(T) 9:11 (C) 10:10	Sodium valproate, acupuncture	Sodium valproate	NM	NM	Effective rate
Zhou et al., 2021	RCT	25:25	T: 32.48 ± 2.19 C: 30.13 ± 2.11	T: 12.39 ± 0.39 C: 12.42 ± 0.41	(T) 13:12 (C) 14:11	Topiramate, acupuncture, dingxianwan decoction	Topiramate	2	6	Effective rate Adverse events
Zhu et al., 2018	RCT	50:50	T: 6.13 ± 2.04 C: 6.22 ± 1.98	T: 4.15 ± 1.26 C: 4.27 ± 1.15	(T) 26:24 (C) 23:27	Sodium valproate, acupuncture	Sodium valproate	6	6	Effective rate Seizure frequency Adverse events
Yu et al., 2015	RCT	30:32	T: 5.51 ± 2.45 C: 5.77 ± 1.59	NM	(T) 18:12 (C) 17:15	Oxcarbazepine, acupuncture	Oxcarbazepine	2	6	Effective rate
Ye et al., 2023	RCT	30:30	T: 44 ± 15 C: 49 ± 15	T: 1.27 ± 0.90 C: 1.70 ± 0.99	(T) 18:12 (C) 19:11	Sodium valproate, acupuncture	Sodium valproate	NM	NM	Effective rate Adverse events EEG discharge frequency QOLIE-31

T, intervention group; C, comparison group; NM, not mention,; M, male; F, female; RCT, randomized controlled trial; EEG, electroencephalogram; QOLIE-31, quality of life: Quality of Life Scale for Epileptic Patients-31; MoCA, the Montreal Cognitive Assessment; MMSE, Mini-mental State Examination; HAMD, Hamilton Depression Scale.

### 3.2. Methodological and reporting quality

**Figure 2:**
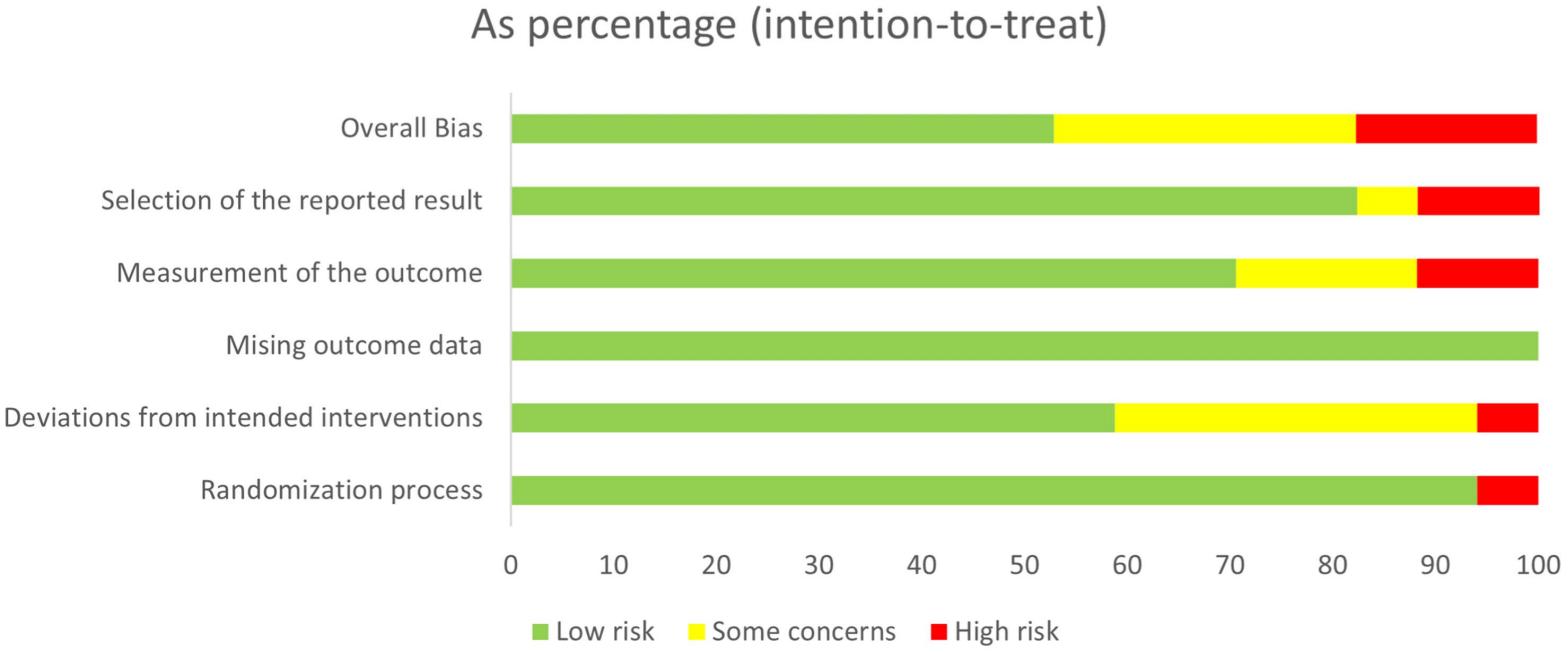
Assessment of risk of bias summary of included studies using the Cochrane tool.

**Figure 3:**
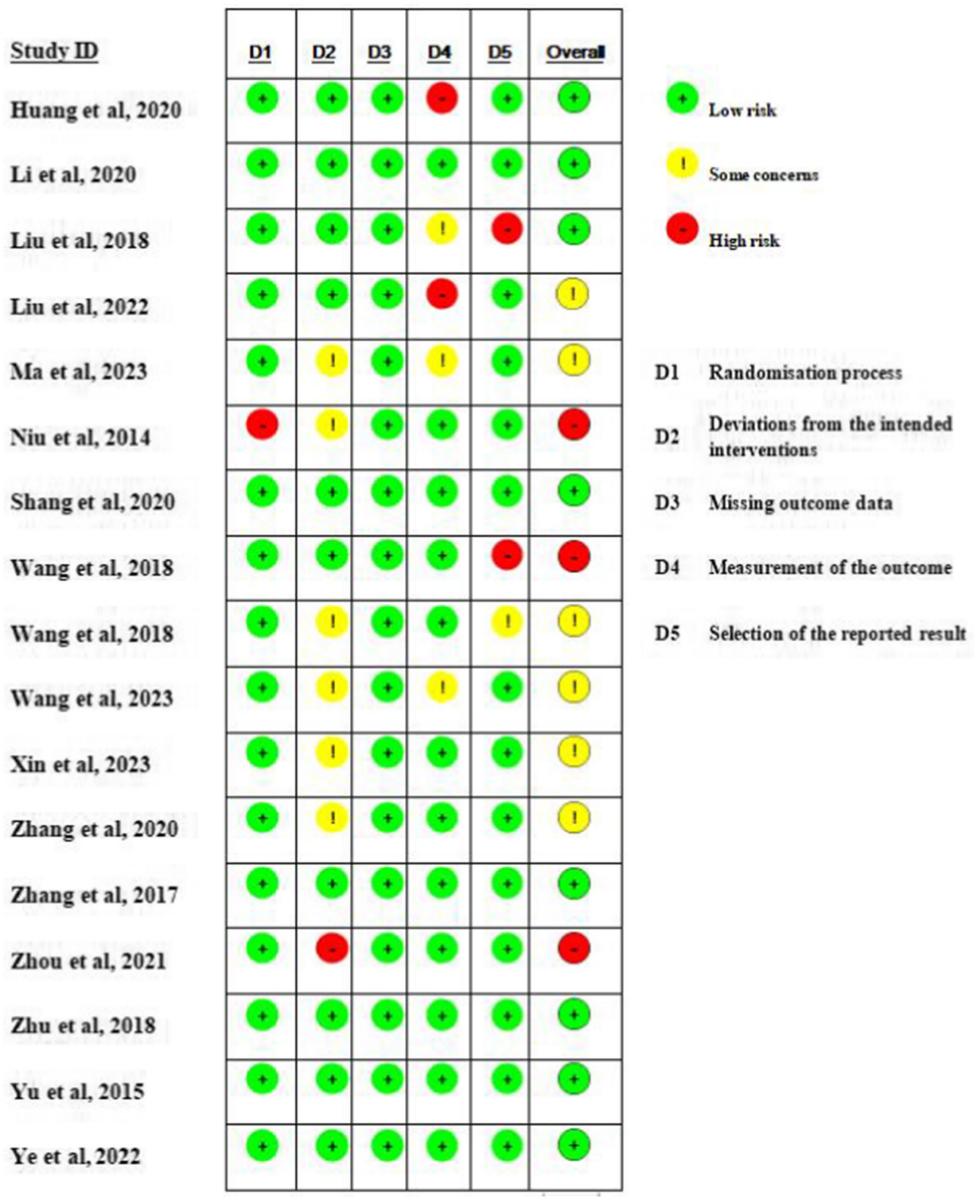
Assessment of risk of bias graph of included studies using the Cochrane tool.

The quality of the 17 included RCTs was generally “low to moderate.” 6 studies (*n* = 6) were considered to be at risk of “some concern,” 8 studies were at low risk, and 3 studies were at high risk of bias. For randomisation process, most studies were at low risk as randomization process was reported in detail, with the technique most commonly used random number tables or computerized allocation methods. One articles only mentioning random were considered “high risk.” For deviations from the intended interventions, ten studies were at low risk, and only one study were at high risk of bias. Detection bias in 2/3 of the studies was rated as being at low risk with two studies being at high risk as their outcome was assessed by non-blinded evaluators. A summary and graph of the ROB assessment are shown in Figures 2, 3.

### 3.3. Effective rate

**Figure 4:**
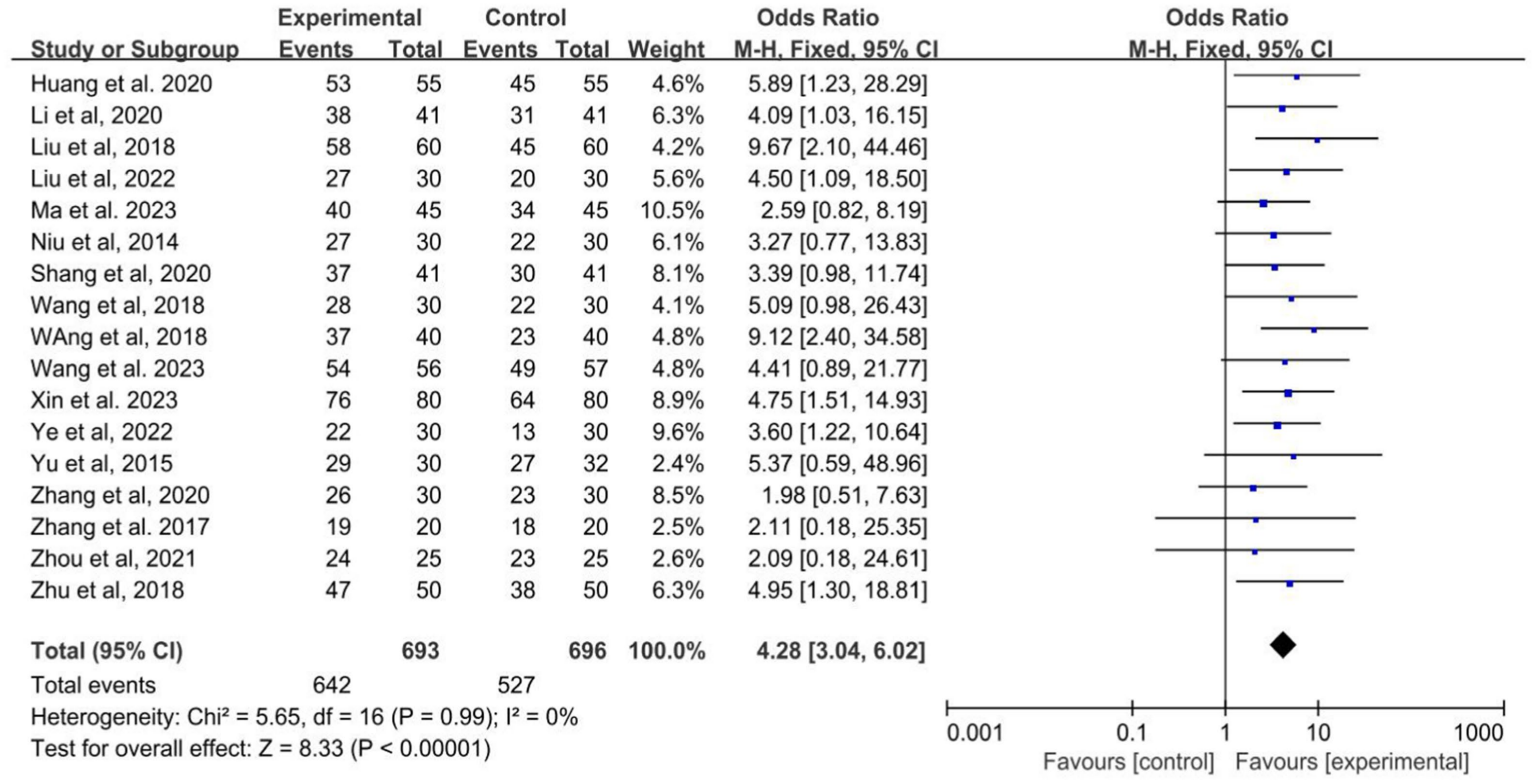
Forest plot of the efficiency of acupuncture combined with Western medicine for epilepsy patients.

The data about the effective rate of acupuncture combined with Western medicine in the treatment of epilepsy were available in 17 studies. The results showed that acupuncture combined with Western medicine improved the effective rates than Western medicine alone (OR: 4.28; 95% CI: 3.04–6.02; *p* < 0.001; Figure 4), and no evidence of heterogeneity using a fixed effects model (*I*^2^ = 0.0%; *p* = 0.99). The pooled conclusion was robust and unaltered by the sequential exclusion of individual trials.

### 3.4. Seizure frequency

**Figure 5:**
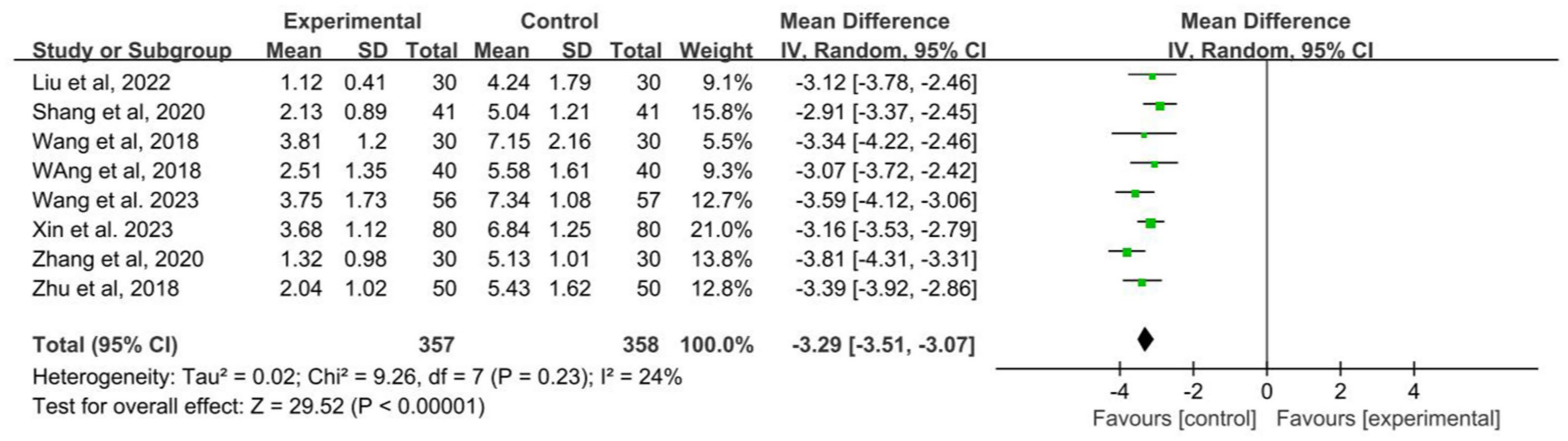
Forest plot of the seizure frequency of acupuncture combined with Western medicine for patients with epilepsy.

Of the 17 studies included, a total of 8 studies recorded seizure frequency (Wang M., 2018; Wang Z. J., 2018; Zhu, 2018; Shang, 2020; Zhang, 2020; Liu, 2022; Wang et al., 2023; Xin et al., 2023). The heterogeneity test suggested mild heterogeneity (*I*^2^ = 24%, *p* = 0.23, Figure 5), using a fixed effect model. The pooled SMD applying a random effect model revealed that acupuncture combined with Western medicine reduces the seizure frequency better than treatment with Western medicine alone (SMD: −3.29; 95% CI: −3.51 to −3.07; *p* < 0.001; Figure 5).

### 3.5. EEG related indicators

#### 3.5.1. EEG discharge frequency

**Figure 6:**
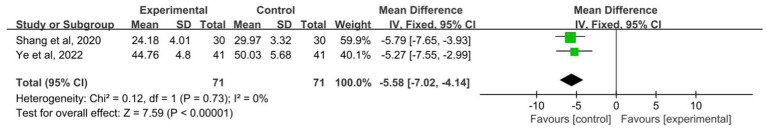
Forest plot of the EEG discharge frequency of acupuncture combined with Western medicine for patients with epilepsy.

Two RCTs assessed the EEG discharge frequency during treatment (Shang, 2020; Ye, 2023), and the combined result of the meta-analysis showed that the combination of acupuncture and Western medicine was more effective than the same medication alone in decreasing the EEG discharge frequency with no evidence of heterogeneity (SMD: −5.58; 95% CI: −7.02 to −4.14; *p* < 0.001; *I*^2^ = 0%, Figure 6).

#### 3.5.2. The power of α wave, β wave, δ wave and θ wave in EEG

A total of three studies assessed the power of α wave, β wave, δ wave and θ wave in EEG (Yu, 2015; Zhang et al., 2017; Wang et al., 2023). The meta-analysis showed that the experimental group was superior to the control group in improving α wave (SMD: 0.58; 95% CI: 0.39–0.78; *p* < 0.001; *I*^2^ = 0%, Supplementary Figure) and β wave (SMD: 0.11; 95% CI: 0.05–0.17; *p* < 0.001; *I*^2^ = 0%, Supplementary Figure), and no evidence of heterogeneity using a fixed effects model. The results of meta-analysis showed that there was no significant difference between the two groups in improving δ wave (SMD: −0.50; 95% CI: −1.20 to 0.20; *p* = 0.16; *I*^2^ = 55%, Supplementary Figure) and θ wave (SMD: 1.12; 95% CI: −1.20–3.45; *p* = 0.34; *I*^2^ = 80%, Supplementary Figure).

### 3.6. QOLIE-31

**Figure 7:**
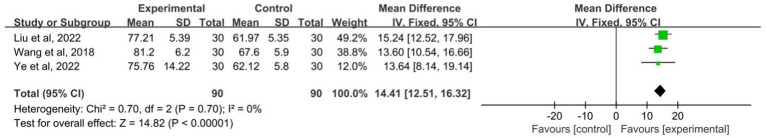
Forest plot of the QOLIE-31 for patient with epilepsy. QOLIE-31, Quality of Life Scale for Epileptic Patients-31.

Only three studies recorded QOLIE-31 scores included 180 patients (Wang M., 2018; Wang Z. J., 2018; Liu, 2022; Ye, 2023). The results showed that the experimental group was superior to the control group in improving the health status and overall quality of life of patients with epilepsy (SMD: 14.41; 95% CI: 12.51–16.32; *p* < 0.001; Figure 7), and no evidence of heterogeneity using a fixed effects model (*I*^2^ = 0.0%, *p* = 0.70, Figure 7).

### 3.7. Adverse events

**Figure 8:**
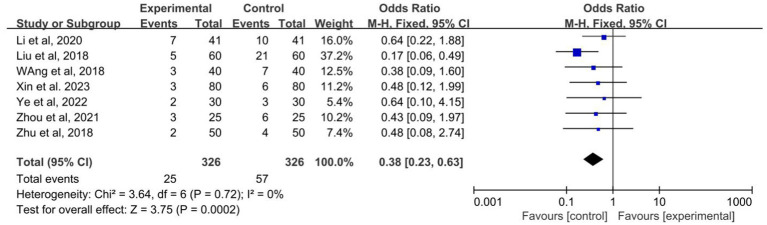
Forest plot of the adverse events of acupuncture combined with Western medicine for patients with epilepsy.

A total of 7 studies recorded the rate of adverse reactions among the included studies (Liu, 2018; Wang M., 2018; Wang Z. J., 2018; Zhu, 2018; Li, 2020; Zhou, 2021; Xin et al., 2023; Ye, 2023). The heterogeneity test was performed: *p* = 0.72, *I*^2^ = 0.0%, which can be considered mild for seven studies heterogeneity, using a fixed effects model. This Meta-analysis showed that OR: 0.38; 95% CI: 0.23–0.63, the difference between the two groups was statistically significant (*p* < 0.01, Figure 8). It can be suggested that the adverse reaction rate of acupuncture combined with the Western medicine group is lower than control group.

### 3.8. Publication bias

**Figure 9:**
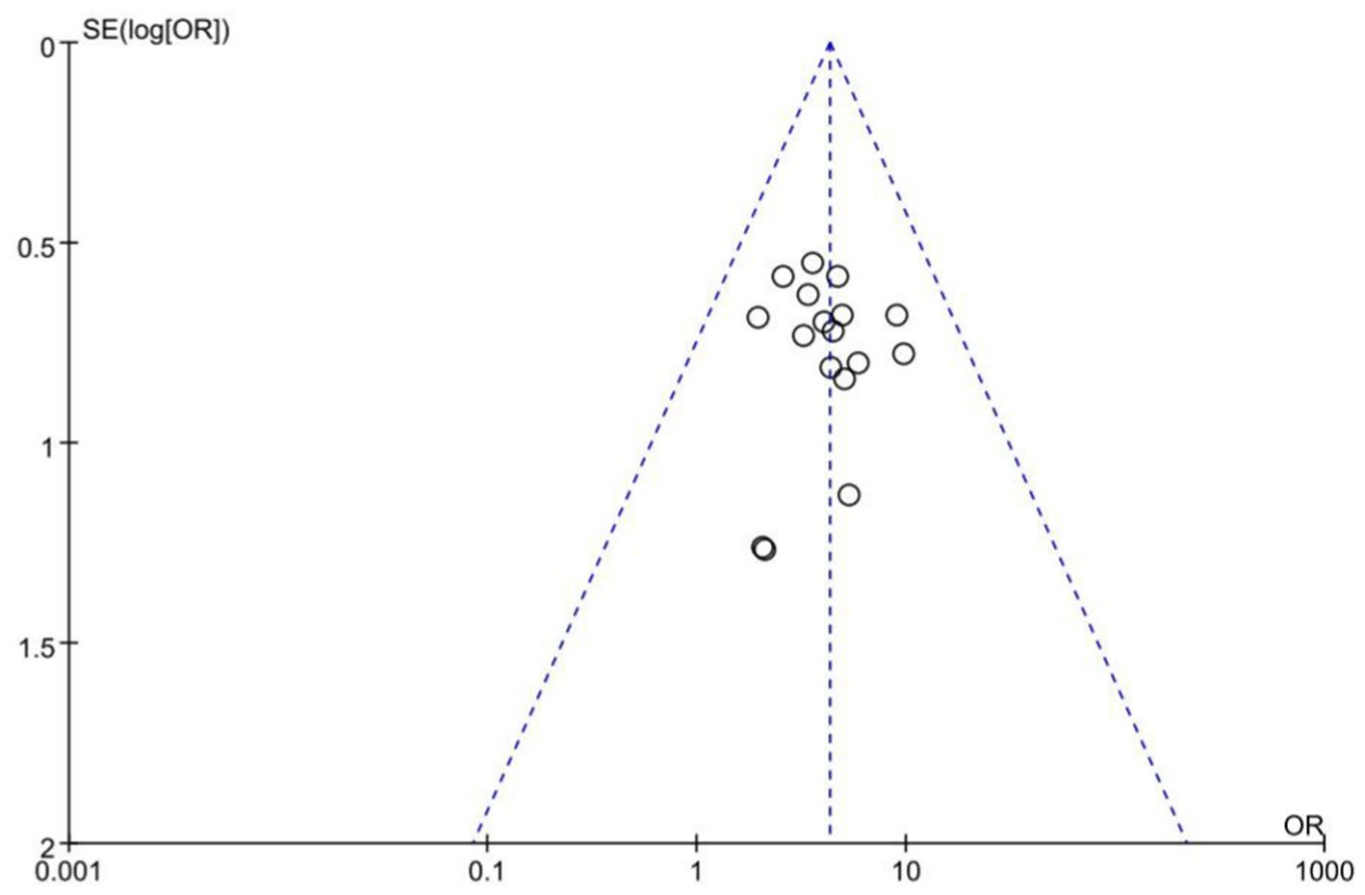
Funnel plot of included studies.

Publication bias assessments are presented as funnel plots (Figure 9). From the roughly symmetrical shapes of these funnel plots, no obvious publication bias was observed.

## 4. Discussion

### 4.1. Summary of main results

This is the first systematic review and meta-analysis conducted based on RCTs for acupuncture combined with Western medicine treatment for epilepsy and assessed the treatment effectiveness of acupuncture as an auxiliary combined with Western medicine on the effective rate, seizure frequency, QOLIE-31, and adverse events for patients with epilepsy. A total of 1,389 patients with primary epilepsy from 17 RCTs, with a broad range of patient characteristics, were included. This meta-analysis have shown that the use of acupuncture could significantly improve the effective rate than conventional therapy. Moreover, acupuncture combined with Western medicine can effectively reduce seizure frequency, decreased the EEG discharge frequency, improving α wave and β wave, and improve the quality of life for patients with epilepsy. In adverse events, adverse reactions were observed in the experimental group and control group. The adverse reactions mainly included dizziness, nausea, vomiting, and diarrhea, and there were no serious adverse reactions between the two groups. This meta-analysis revealed that the control group is more prone to adverse reactions than the experimental group.

There were no systematic reviews and meta-analyses that addressed the treatment effectiveness of acupuncture combined with Western medicine in patients with epilepsy. At present, acupuncture for epilepsy is widely used in clinical practice, and most of them report reliable efficacy, indicating that acupuncture for epilepsy has certain advantages and development potential (Zhong, 2014; Zhao et al., 2022). Throughout the papers published in recent years on the treatment of epilepsy with acupuncture, it can be found that a variety of acupuncture therapies, including acupoint embedding and electroacupuncture, have obvious effects, and have the advantages of simple operation, economic and inexpensive and long duration of therapeutic effects, which have obvious advantages in the treatment of epilepsy. This has been verified by many researchers through clinical studies (Zhu, 2015).

Xue et al. animal experimental model study suggested that acupuncture at Zusanli point can improve the degree of epileptic seizures in epileptic rats, reduce the damage of hippocampal nerve cells, and play a role in the treatment of epilepsy. The mechanism may be related to the reduction of the expression of leptin, chemotactic factor, and immune cytokines immune gamma globulins 1 (IgG1) and immune gamma globulins 2 (IgG2) in the hippocampus. Under different functional states of rats, Zusanli point has specific and selective effects on hippocampal cell morphology, apoptosis, and cytokine expression: acupuncture at Zusanli point has specific bidirectional effects on the expression of hippocampal CA1 and dentate gyrus cells. It has a specific regulatory effect on early cell apoptosis under normal conditions and a specific regulatory effect on the expression of cytokines chemotactic factor and immunoglobulin globulins 2a (IgG2a) under epileptic conditions (Xue, 2018).

Despite a large number of case reports that acupuncture is effective in the treatment of epilepsy, however, there are also clinical observational studies that have shown inconclusive results of acupuncture in the treatment of epilepsy. Stavem et al. published a clinically controlled study of 29 patients with chronic intractable epilepsy. They assessed the efficacy in terms of seizure frequency (Stavem et al., 2000). The patients were randomly divided into 2 groups: 15 with traditional acupuncture and 14 with sham acupuncture. It was found that seizure frequency was reduced in both groups, but the difference was not statistically significant when compared between the 2 groups. They therefore concluded that the effectiveness of acupuncture in chronic intractable epilepsy is uncertain. We expect that additional high-quality RCTs with placebo controlled trials examining the use of acupuncture as a treatment for epilepsy involving larger sample sizes, more outcomes, and longer observation periods will be conducted to assess the clinical value and safety of acupuncture.

### 4.2. Strengths and limitations

This meta-analysis has several limitations. Firstly, the quality of the included literature is unsatisfied, 17 RCTs are from China and no relevant literature from other countries has been published, which may lead to bias. Secondly, some of the literature has bias in the design method in terms of incomplete description of the randomized method, blinded method and allocation concealment not mentioned. Thirdly, the implementation process of acupuncture is somewhat subjective and not completely under the same criteria, such as the selection of acupoints and the manipulation of techniques, which can lead to different efficacy. Therefore, there may be implementation bias and publication bias in this study, and these biases may have an impact on the results of the meta-analysis.

## 5. Conclusion

In conclusion, the results of this study demonstrated that acupuncture combined with Western medicine may have advantages in the treatment of epilepsy, which improve the effective rate, and quality of life for patients with epilepsy. However, the quality of the included studies is generally low to moderate, and the conclusions need to be viewed with caution. It is believed that with the continuous enrichment of clinical research data and the addition of evidence-based medical evidence, the diagnosis and treatment of epilepsy will be further improved, and eventually the most optimal treatment plan will be brought to epilepsy patients to achieve the best treatment effect.

## Author contributions

HX and LZ carried out the conception and design of the research and drafted the manuscript. HX and DX participated in the acquisition of data. HX carried out the analysis and interpretation of data. HX and HH performed the statistical analysis. DX participated in the revision of the manuscript for important intellectual content. All authors contributed to the article and approved the submitted version.

## Conflict of interest

The authors declare that the research was conducted in the absence of any commercial or financial relationships that could be construed as a potential conflict of interest.

## Publisher’s note

All claims expressed in this article are solely those of the authors and do not necessarily represent those of their affiliated organizations, or those of the publisher, the editors and the reviewers. Any product that may be evaluated in this article, or claim that may be made by its manufacturer, is not guaranteed or endorsed by the publisher.
