# Supplementary material for: Effectiveness of acupuncture as auxiliary combined with Western medicine for epilepsy: a systematic review and meta-analysis
Source: Front Neurosci. 2023 Jul 20;17:1203231. doi: 10.3389/fnins.2023.1203231 (PMC10397512; doi:10.3389/fnins.2023.1203231)
Supplement: Supplementary file 1 [file Data_Sheet_1.docx]

Supplementary Material

Effectiveness of acupuncture as auxiliary combined with Western medicine for epilepsy: A systematic review and Meta-analysis

Hua Xue^1*^, Li Zeng^2^, Hongxian He^1^, Dongxun Xu^1^, Kaixin Ren^3^

^1^Department of Neurology, Sichuan Taikang Hospital, Chengdu, Sichuan, 610213, China

^2^Department of Respiratory, Affiliated Hospital of Youjiang Medical University for Nationalities,

Baise, Guangxi, 533000, China

^3^Department of Rehabilitation, Affiliated Hospital of Yunnan University, Kunming, Yunnan, 650032, China

*** Correspondence:** Hua Xue: [xueh1895@163.com](mailto:xueh1895@163.com)

**Supplementary Materials**

**Search strategy**

| PUBMED | |
| --- | --- |
| #1 | Epilepsy [Mesh] |
| #2 | ((((((Epilepsies[Title/Abstract]) OR (Seizure Disorder[Title/Abstract])) OR (Seizure Disorders[Title/Abstract])) OR (Awakening Epilepsy[Title/Abstract])) OR (Epilepsy, Awakening[Title/Abstract])) OR (Cryptogenic Epilepsies[Title/Abstract])) OR (Auras[Title/Abstract]) |
| #3 | #1 OR #2 |
| #4 | Acupuncture Therapy[Mesh] |
| #5 | (((((((((Acupuncture Treatment[Title/Abstract]) OR (Acupuncture Treatments[Title/Abstract])) OR (Treatment, Acupuncture[Title/Abstract])) OR (Therapy, Acupuncture[Title/Abstract])) OR (Pharmacoacupuncture Treatment[Title/Abstract])) OR (Treatment, Pharmacoacupuncture[Title/Abstract])) OR (Pharmacoacupuncture Therapy[Title/Abstract])) OR (Therapy, Pharmacoacupuncture[Title/Abstract])) OR (Acupotomy[Title/Abstract])) OR (Acupotomies[Title/Abstract]) |
| #6 | #4 OR #5 |
| #7 | # 6 AND #3 |
| Embase | |
| #1 | 'epilepsy'/exp |
| #2 | 'epilepsy':ab,ti |
| #3 | ##1 OR #2 |
| #4 | 'acupuncture'/exp |
| #5 | 'acupressure':ab,ti |
| #6 | 'needle':ab,ti |
| #7 | 'electroacupuncture':ab,ti |
| #8 | #4 OR #5 OR #6 OR #7 |
| #9 | #8 AND #3 |
| Cochrane library | |
| #1 | MeSH descriptor: [Acupuncture] explode all trees |
| #2 | (Acupuncture Treatment):ti,ab,kw OR (Acupuncture Treatments):ti,ab,kw OR (Treatment, Acupuncture):ti,ab,kw OR (Acupotomy):ti,ab,kw OR ("needle"):ti,ab,kw |
| #3 | #1 OR #2 |
| #4 | MeSH descriptor: [epilepsy] explode all trees |
| #5 | (epilepsy):ti,ab,kw OR (Seizure):ti,ab,kw OR (Seizure Disorder):ti,ab,kw |
| #6 | #4 OR #5 |
| #7 | #6 AND #3 |
| China National Knowledge Infrastructure (CNKI) | |
| ( SU='针灸' OR SU='针刺' OR SU='电针' OR SU='头针' OR SU='耳针' OR SU=温针' OR SU='浮针' OR SU='眼针' OR SU='皮内针' OR SU='火针' OR SU='温针' OR SU='体针' OR SU='腕踝针' OR SU='干针' OR SU='芒针' ) AND (SU='癫痫' OR SU='原发性癫痫'）AND (TKA='随机' OR TKA='对照') | |
| Wangfang Database | |
| (((((主题=癫痫) OR 主题=继发性癫痫))) AND ((((((主题=针灸) OR 主题=针刺) OR 主题=体针) OR 主题=温针灸) OR 主题=电针))) | |
| Chinese BioMedical Literature Database | |
| 1. "癫痫"[不加权:扩展] 2. "癫痫"[加权:扩展] OR "原发性癫痫"[加权:扩展] OR "羊角风"[加权:扩展] OR "痹症"[加权:扩展] OR "羊癫疯"[加权:扩展] 3. "针灸"[加权:扩展] OR "体针"[加权:扩展] OR "温针"[加权:扩展] OR "火针"[加权:扩展] OR "穴位"[加权:扩展] OR "温针灸"[加权:扩展] 4. ((#3) AND (#2)) | |

***Supplementary Figure***

***
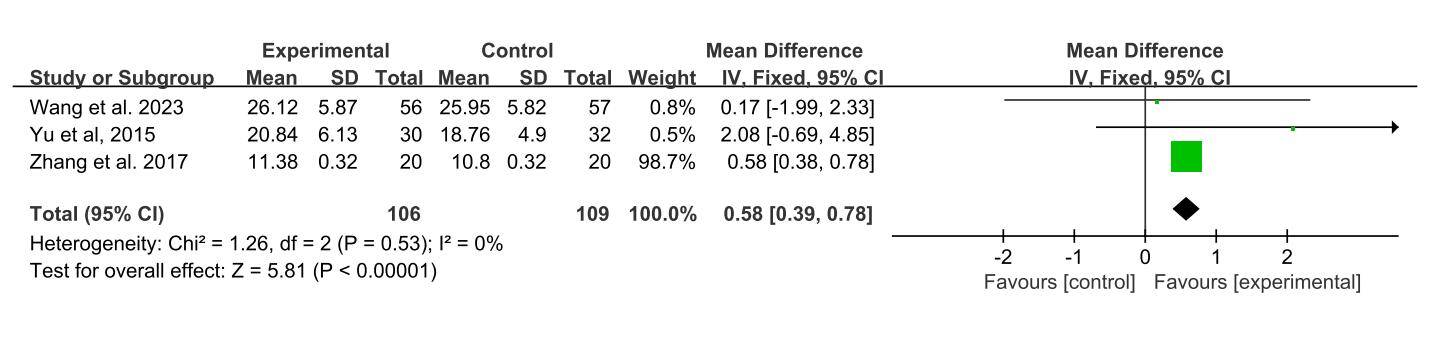
***

Forest plot of the α wave of acupuncture combined with Western medicine for patients

with epilepsy


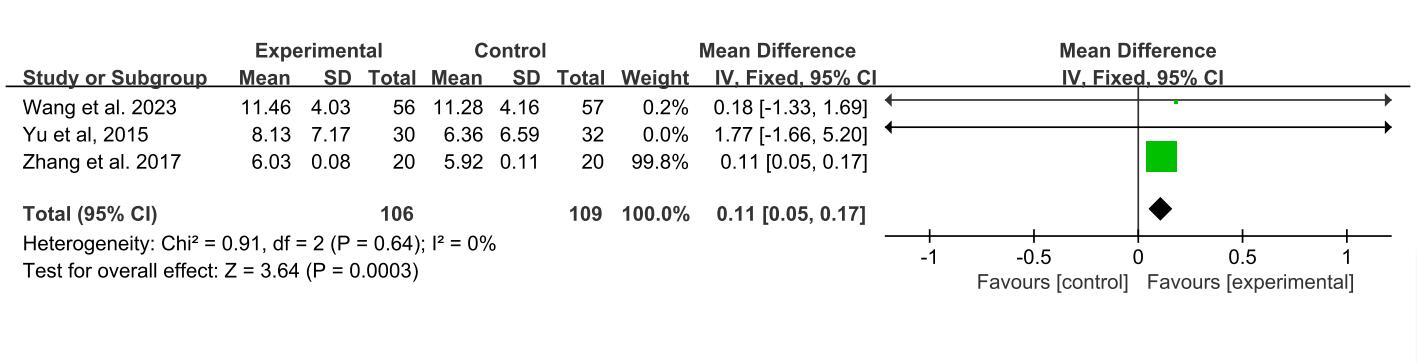
Forest plot of the β wave of acupuncture combined with Western medicine for patients

with epilepsy

Forest plot of the δ wave of acupuncture combined with Western medicine for patients

with epilepsy


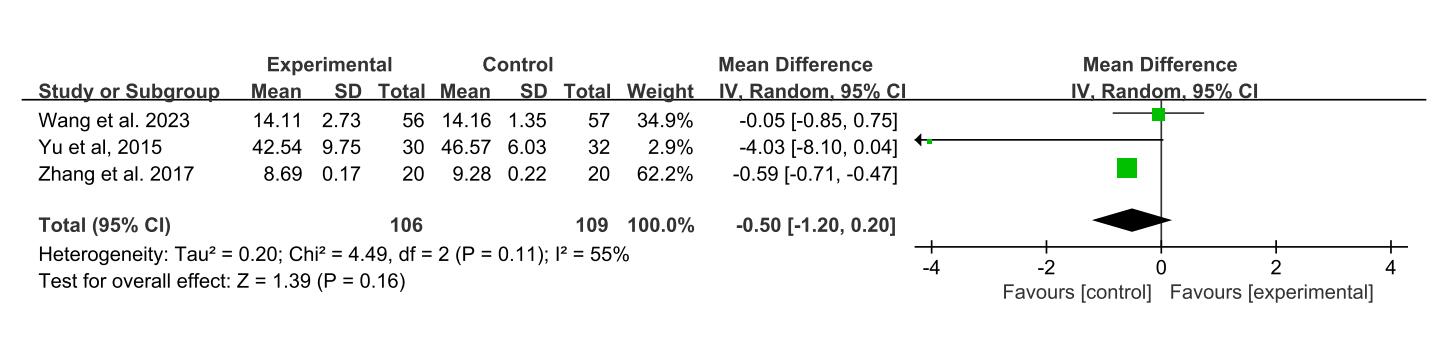


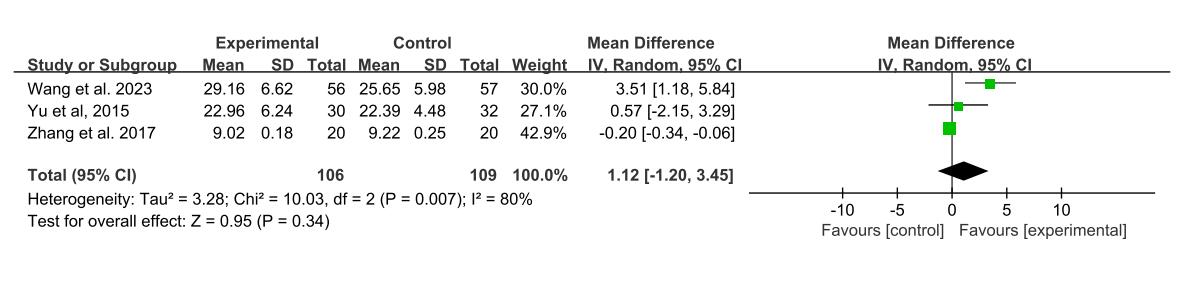


Forest plot of the θ wave of acupuncture combined with Western medicine for patients

with epilepsy
